# Genome-wide analysis of the *CaHsp20* gene family in pepper: comprehensive sequence and expression profile analysis under heat stress

**DOI:** 10.3389/fpls.2015.00806

**Published:** 2015-10-01

**Authors:** Meng Guo, Jin-Hong Liu, Jin-Ping Lu, Yu-Fei Zhai, Hu Wang, Zhen-Hui Gong, Shu-Bin Wang, Ming-Hui Lu

**Affiliations:** ^1^Department of Vegetable Science, College of Horticulture, Northwest A&F UniversityYangling, China; ^2^Laboratory for Solanaceous Fruit Vegetable, Institute of Vegetable Crops, Jiangsu Academy of Agricultural SciencesNanjing, China

**Keywords:** *Capsicum annuum* L., *CaHsp20 genes*, heat stress, expression profile, gene family

## Abstract

The *Hsp20* genes are present in all plant species and play important roles in alleviating heat stress and enhancing plant thermotolerance by preventing the irreversible aggregation of denaturing proteins. However, very little is known about the *CaHsp20* gene family in pepper (*Capsicum annuum* L.), an important vegetable crop with character of temperate but thermosensitive. In this study, a total of 35 putative pepper *Hsp20* genes (*CaHsp20s*) were identified and renamed on the basis of their molecular weight, and then their gene structure, genome location, gene duplication, phylogenetic relationship, and interaction network were also analyzed. The expression patterns of *CaHsp20* genes in four different tissues (root, stem, leaf, and flower) from the thermotolerant line R9 under heat stress condition were measured using semi-quantitative RT-PCR. The transcripts of most *CaHsp20* genes maintained a low level in all of the four tissues under normal temperature condition, but were highly induced by heat stress, while the expression of *CaHsp16.6b, 16.7*, and *23.8* were only detected in specific tissues and were not so sensitive to heat stress like other *CaHsp20* genes. In addition, compared to those in thermotolerant line R9, the expression peak of most *CaHsp20* genes in thermosensitive line B6 under heat stress was hysteretic, and several *CaHsp20* genes (*CaHsp16.4, 18.2a, 18.7, 21.2, 22.0, 25.8*, and *25.9*) showed higher expression levels in both line B6 and R9. These data suggest that the *CaHsp20* genes may be involved in heat stress and defense responses in pepper, which provides the basis for further functional analyses of *CaHsp20s* in the formation of pepper acquired thermotoleance.

## Introduction

As the sessile organisms, plants cannot escape the extreme environment conditions, including biotic and abiotic stresses, such as pests and pathogens, drought, salinity, cold, heat, and heavy metal stresses (Wang et al., 2003; Mittler, 2006; Mittler et al., 2012). Increase in the global warming having adverse effects of heat stress (HS) on crop plants growth, development and productivity are more serious, thus plants adaptive response to high temperature i.e., hypersensitive response (HSR) is very important for world food security (Glazebrook, 2001; Giorno et al., 2010). Under HS conditions, the heat shock transcription factors (Hsfs) bind to the heat shock elements (HSEs) of the promoters of heat-responsive genes, mainly the heat shock proteins (Hsps), to turn on or regulate their expression (Scharf et al., 2012; Waters, 2013). Plant Hsps as molecular chaperones are involved in maintaining homeostasis of protein folding, preventing or repairing the misfolding and degradation of proteins caused by HS, and then confer the acquisition of plant thermotolerance (Vierling, 1991; Sung et al., 2003; Charng et al., 2006).

The plant Hsps are grouped into five families based on the molecular weight and sequence homology, including the Hsp100s, Hsp90s, Hsp70s, Hsp60s, and Hsp20s (Sarkar et al., 2009; Waters, 2013). Among all the five conserved families, Hsp20s, a group of small heat shock proteins (sHsps) with molecular size ranging from 15 to 42 kDa, are the most prevalent and abundant family induced by HS in many higher plants. Furthermore, they can function as molecular chaperone without ATP to maintain the stability of proteins, which suggest that Hsp20s may play a vital role during the formation of plant acquired thermotolerance (Vierling, 1991; Sun et al., 2002; Wang et al., 2004; Waters, 2013; Haslbeck and Vierling, 2015).

Hsp20s share a conserved modular structure, and the most striking feature is a core conserved alpha-crystallin domain (ACD, the Hsp20 domain) containing ~90 amino acids flanked by a variable N-terminal domain and a C-terminal extension (Vierling, 1991; Scharf et al., 2001; Waters, 2013), in which the ACD is involved in substrate interactions, the N-terminus is responsible for substrate binding and the C-terminal participates in homo-oligomerization and the formation of HS granula (Kirschner et al., 2000; Giese and Vierling, 2004; Basha et al., 2006; Jaya et al., 2009). The ACD is composed of two anti-parallel sheets of four and three β-strands, i.e., conserved regions I (CRI, β2-β3-β4-β5) in N-terminal consensus and CRII (β7-β8-β9) in C-terminal consensus, respectively, connected by a hydrophobic region loop (β6-loop; Bondino et al., 2012; Haslbeck and Vierling, 2015). Besides, the N-terminus contains the transit, targeting or signal sequences, and the C-terminal may also contain organelle-specific retention amino acid motifs (Waters, 2013).

The Hsp20s form a more diverse family than other four Hsp families in plants, and are also far more than the members of Hsp20 families in animals (Wang et al., 2004; Waters, 2013). Plant Hsp20s are encoded by nuclear genes and are divided into various subfamilies based on cellular location, sequence homology and function (Vierling, 1991; Waters et al., 1996). Nineteen *Hsp20* genes in *Arabidopsis* (Scharf et al., 2001; Waters, 2013), 23 in *Oryza sativa*, 36 in *Populus trichocarpa* (Waters et al., 2008), and 51 in *Glycine max* were identified, furthermore, the *Hsp20* genes in *Arabidopsis* were grouped into 11 subfamilies (Scharf et al., 2001; Waters, 2013), while those from *G. max* were grouped into 15 subfamilies based on phylogenetic analysis, prediction of subcellular localization and prediction of protein secondary structure models (Lopes-Caitar et al., 2013). However, to our knowledge, genome-wide analysis of *Hsp20* family in pepper is still lacking.

Pepper (*Capsicum annuum* L.) is an important vegetable crop worldwide, and is deeply loved by a large population due to its major ingredient in cuisines, essential vitamins and other healthy nutrients (Kim et al., 2014). However, pepper is sensitive to high temperature, especially in reproductive stage (Guo et al., 2014). Once suffering continuous HS, the pollination and fertilization of pepper will be severely damaged, which can greatly reduce the yield and quality of pepper fruits. Therefore, study of Hsp20s is important for understanding the mechanism of heat response in pepper, while except several *CaHsp20* genes, such as *CaHsp24* (Zhu et al., 2011), *CaHsp26* (Guo et al., 2007), little is currently known about the *C. annuum* heat shock protein 20 (CaHsp20) family.

In this study, we identify and analyze 35 *CaHsp20* genes from pepper genome, focusing on their heat-induced expression in various tissues, and their dynamic expression patterns in two different thermotolerant pepper lines under HS condition. Our results provide a foundation for further functional analysis of *CaHsp20* members, and will be valuable for illuminating the mechanism of pepper acquired thermotolerance, and even the adaptability of pepper to other stress conditions.

## Materials and methods

### Genome-wide identification of *CaHsp20* genes in pepper

The Hsp20 proteins are defined by possessing a highly conserved 80–100 amino acid residues called ACD, and a low molecular weight (15–42 kDa; Vierling, 1991; Scharf et al., 2001). To identify the Hsp20 family members in pepper, the Hidden Markov Model (HMM) profile of ACD (PF00011) downloaded from the Pfam protein family database (http://pfam.sanger.ac.uk) was used as queries to search the Pepper Genome Database (PGD, http://peppergenome.snu.ac.kr/, CM334, Zunla-1 and glabriusculum genomes; Kim et al., 2014; Qin et al., 2014). Secondly, all *O. sativa* and *G. max* Hsp20 protein sequences (Ouyang et al., 2009; Lopes-Caitar et al., 2013) were used as query sequences to search against the PGD and the NCBI database. All output putative pepper *Hsp20* genes with an *e*-value ≤0.001 were assembled to remove redundancy, and then submitted to Pfam and SMART (http://smart.embl-heidelberg.de/) to confirm the presence of the ACD domain. The Multiple Sequence Alignment (MSA) was subsequently trimmed in order to remove subsequences that were not truly homologous. The predicted genes lacking an ACD domain and/or the genes whose molecular weight out of the range from 15 to 42 kDa were rejected. All non-redundant and confident genes were assigned as pepper *Hsp20* genes (*CaHsp20s*), and annotated based on their molecular weight as with soybean (Lopes-Caitar et al., 2013). The logic flowchart of identification of pepper *Hsp20* genes was shown in Figure S1.

Using the same method, the putative *Hsp20* homologs in tomato, *Arabidopsis*, rice, and maize were obtained from the SOL Genomics Network (SGN) database (http://solgenomics.net/organism/Solanum_lycopersicum/genome), the TAIR database (https://www.arabidopsis.org/index.jsp), the Rice Genome Annotation Project (http://rice.plantbiology.msu.edu/), and the TIGR Maize Database (http://maize.jcvi.org/), respectively.

The protein sequences of putative CaHsp20 members were analyzed with EXPASY PROTOPARAM (http://www.expasy.org/tools/protparam.html) to obtain the number of amino acids, molecular weight, theoretical isoelectric point (pI) and instability index (with a value >40 considered as unstable; Lopes-Caitar et al., 2013). The chromosomal location data and intron numbers of putative *CaHsp20s* were retrieved from PGD. The WoLF PSORT program (http://wolfpsort.org/) and TargetP (Emanuelsson et al., 2000) were used for predicting the subcellular locations.

### Analysis of conserved motifs of pepper *CaHsp20* family

The conserved domains of the CaHsp20 proteins (ACD) were confirmed by Pfam and SMART. The MEME program (Version 4.10.0, http://meme.nbcr.net/meme/tools/meme) was used for identification of the conserved motifs, with the following parameters: number of repetitions-any, maximum number of motifs-10, the optimum motif widths set from 6 to 200 amino acid residues; the conserved motifs were annotated with InterProScan (http://www.ebi.ac.uk/interpro/search/sequence-search; Ouyang et al., 2009). The ACD sequences of all CaHsp20 members were aligned using the Promals3D structural alignment program (http://prodata.swmed.edu/promals3d/promals3d.php).

### Phylogenetic analysis and classification of pepper *CaHsp20* genes

The full amino acid sequences of Hsp20 members from pepper, tomato, *Arabidopsis*, rice, and maize were aligned by CLUSTALW program. The gene IDs of *Hsp20* members from tomato, *Arabidopsis*, rice, and maize were shown in Table S1. An un-rooted neighbor-joining phylogenetic tree was constructed using MEGA 5.10 software (Tamura et al., 2011) with the bootstrap test replicated 1000 times, pair wise deletion and a Poisson model. The pepper *CaHsp20* genes were assigned to different groups based on the multiple sequence alignment and the classification of *Hsp20* genes in the other four species.

### Chromosomal localization and gene duplication

Each of the *CaHsp20* genes was mapped on chromosomes using MapDraw (Liu and Meng, 2003) based on their chromosomal position and the relative distance between these genes on the same chromosome derived from PGD. The *CaHsp20* gene duplication was defined based on the following criteria: (1) the length of the sequence alignment covered ≥70% of the longer gene; and (2) the similarity of the aligned gene regions ≥70% (Gu et al., 2002; Yang et al., 2008). Genes separated by five or fewer genes in a 100-kb region were considered to be tandem duplicates (Wang et al., 2010); genes were regarded as segmental duplication if they were found to be coparalogs located on duplicated chromosomal blocks, as proposed by Wei et al. (2007).

### Prediction of CaHsp20s protein–protein interaction network

An interaction network of CaHsp20 proteins from pepper was constructed to understand the genome-wide regulation network; the interolog from *Arabidopsis* was used for predicting protein–protein interaction network of CaHsp20 members. An interaction work of *Arabidopsis* Hsp20 proteins was constructed using the *Arabidopsis* Interactions Viewer (http://bar.utoronto.ca/interactions/cgi-bin/arabidopsis_interactions_viewer.cgi). *Arabidopsis* Hsp20 proteins were mapped to pepper CaHsp20 proteins based on their homologous relationship, and an interaction network of CaHsp20 proteins was drawn by Cytoscape_v2.8.3 (National Institute of General Medical Sciences, MD, USA).

### Plant materials and heat stress treatments

The pepper thermotolerant line R9 and the thermosensitive line B6 were used in this study. Both lines were grown in a growth chamber at 26°C (day for 16 h)/20°C (night for 8 h) till reaching the age of 6–8 true leaves. R9 plants were used for the tissue specific expression analysis, the seedlings with 6–8 true leaves and the plants with flower buds were treated with HS of 40°C for 2 h by placing in a light incubator (GXZ-380C, Jiangnan Instrument Factory, Ningbo, China) and others under 26°C for 2 h were used as the control. The young leaves, stems and roots from seedling plants and the flower buds from adult plants with or without HS treatments were collected. For the dynamic expression analysis of *CaHsp20s* under HS, after being subjected to 40°C for 0, 0.5, 1, 2, 4 h, the leaves in B6 and R9 were collected, respectively. Temperature treatment was initiated during the light period and all the samples were harvested within the light period. These collected samples were frozen with liquid nitrogen for total RNA extraction and cDNA synthesis. Each treatment was conducted with three biological replicates, and samples from five plants were collected for each replicate.

### RNA isolation and expression analysis of pepper *CaHsp20* genes

Total RNA was extracted using Total RNA kit (BioTeke, Beijing, China) and reverse-transcribed using SuperScriptIII Reverse Transcriptase (Takara, Dalian, China), the operational procedure followed the manufacturer's procedure, and the cDNA was diluted to 50 ng/μL with ddH_2_O for further examination. For expression analysis of pepper *CaHsp20s* in different tissues, semi-quantitative reverse-transcription (RT) PCR reactions were employed. Primer pairs (Table S2) were designed by Primer Premier 5.0 and tested by NCBI Primer BLAST. Ubiquitin binding protein gene *CaUBI-3* was used as the reference genes (Wan et al., 2011). PCR reactions for all genes were conducted with the following program: initial denaturation at 94°C for 5 min, followed with denaturation at 94°C for 30 s, annealing at 52°C for 30 s, extension at 72°C for 30 s with 30 cycles, and final extension at 72°C for 10 min. PCR products were detected by agarose gel electrophoresis with 1.5% gel concentration. The expression levels of *CaHsp20* genes in each sample were compared with the internal control gene, *CaUBI-3*.

Real-time quantitative PCR (qRT-PCR) was conducted using SYBR Green Supermix (Takara, Dalian, China) with a total volume of 20 μL reaction system on the iQ5.0 Bio-Rad iCycler thermocycler (Bio-Rad, Hercules, CA, USA). The primers of *CaHsp20* genes were the same as those used for semi-quantitative RT-PCR. The qRT-PCR reactions were performed as described (Guo et al., 2014). The *CaUBI-3* gene was used as the internal reference gene. Three independent biological replicates were carried out and qRT-PCR of each replicate was performed in triplicate. The relative expression levels of pepper *Hsp20* genes were calculated as 2^−ΔΔCT^ method (Schmittgen and Livak, 2008).

### Search for *cis*-acting elements in the promoters of pepper *CaHsp20* genes

The upstream regions (0.5 kb) of the *CaHsp20* genes were derived from PGD, and were searched for regulatory elements, including HSEs (*cis*-acting element involved in heat stress responsiveness), TATA-box (core promoter element around −30 of transcription start), TC-rich repeats (*cis*-acting element involved in defense and stress responsiveness), W-box (binding site for the WRKY transcription factor in defense response), and CCAAT sequences (enhancer-binding protein factors) in the promoters were performed in PlantCARE (http://bioinformatics.psb.ugent.be/webtools/plantcare/html/; Lescot et al., 2002) and PLACE database (Higo et al., 1999).

## Results

### Identification and analysis of *CaHsp20* gene family members in pepper

A total of 41 sequences of *Hsp20* gene were gotten from PGD by HMM analysis, out of which 35 were annotated as being pepper *Hsp20* genes based on the confirmation of Pfam and SMART scans, and sequences without a complete domain (*CA09g15370, CA12g15460*, and *CA03g18690*) and sequences whose molecular weight out of the range of Hsp20s (*CA08g09970, CA04g23450*, and *CA00g46140*) were removed. The 35 *CaHsp20* genes were named according to their molecular weight (Table 1).

**Table 1:** **The list of ***CaHsp20*** members identified**.

**No**.	**Name**	**Gene ID**	**Chr**.	**ORF**	**Introns**	**AA**	**ACD**	**WT**	**pI**	**Localization predicted**	**Instability index**
1	CaHsp16.3	CA05g01890	5	444	1	147	29–144	16.33	6.84	cyto, mito	47.58
2	CaHsp16.4	CA02g30320	2	426	1	141	29–138	16.35	8.47	Secretory pathway	33.80
3	CaHsp16.6a	CA03g37070	3	447	1	148	40–145	16.55	5.79	cyto, extr	65.53
4	CaHsp16.6b	CA10g22470	10	435	1	144	16–97	16.55	9.04	cyto, extr, E.R.	14.10
5	CaHsp16.7	CA04g23370	4	462	1	153	35–130	16.66	6.29	chlo, extr	47.17
6	CaHsp17.2	CA09g08950	9	447	0	148	38–147	17.22	5.66	cyto	37.04
7	CaHsp17.6	Capana06g000385▴	6	465	0	154	50–153	17.60	5.83	cyto	49.55
8	CaHsp17.7a	Capana03g001238▴	3	477	0	158	52–156	17.73	6.17	cyto, nucl	39.33
9	CaHsp17.7b	Capana03g001241▴	3	477	0	158	52–156	17.68	6.75	cyto, nucl	36.08
10	CaHsp17.8a	CA06g24940	6	465	0	154	50–153	17.76	5.83	cyto	52.77
11	CaHsp17.8b	CA06g24930	6	465	0	154	50–153	17.78	6.00	cyto	53.63
12	CaHsp18.1a	CA08g17060	8	480	0	159	55–158	18.12	5.58	cyto	68.08
13	CaHsp18.1b	CA08g17070	8	480	0	159	55–158	18.09	5.84	cyto	71.86
14	CaHsp18.2a	CA08g17040	8	480	0	159	55–158	18.22	5.39	cyto, nucl	69.52
15	CaHsp18.2b	CA08g17050	8	480	0	159	55–157	18.18	8.83	cyto	58.82
16	CaHsp18.4a	Capana00g002751▴	4	510	1	169	64–168	18.43	5.07	cyto	56.93
17	CaHsp18.4b	Capana01g000571▴	1	513	1	170	89–163	18.43	5.68	chlo	37.86
18	CaHsp18.7	CA06g20260	6	486	1	161	66–160	18.73	5.08	Secretory pathway	58.55
19	CaHsp21.1	Capana06g000831▴	6	561	0	187	71–168	21.12	6.36	Secretory pathway	42.46
20	CaHsp21.2	Capang01g004332⋆	8	558	0	186	72–167	21.20	5.20	Secretory pathway	46.21
21	CaHsp21.7	CA07g18820	7	573	1	190	89–185	21.67	5.19	cyto, pero	38.57
22	CaHsp22.0	CA03g27140	3	579	0	192	70–173	22.03	6.05	Secretory pathway	48.98
23	CaHsp22.1	Capang10g001114⋆	10	585	1	194	96–187	22.11	9.14	chlo	45.05
24	CaHsp22.6	CA05g03530	5	603	0	200	78–186	22.63	5.37	Secretory pathway	38.03
25	CaHsp23.8	CA09g06120	9	612	1	203	102–203	23.83	7.15	cyto, nucl	54.99
26	CaHsp24.2	CA01g05030	1	636	1	211	115–210	24.15	5.18	cyto, chlo, mito	61.58
27	CaHsp24.3a	CA09g08090	9	648	0	215	119–214	24.27	7.86	chlo, cyto, mito	68.39
28	CaHsp24.3b	Capana01g001839▴	1	648	1	215	35–108	24.32	9.21	cyto, golg, nucl	42.06
29	CaHsp25.8	CA03g21390	3	705	1	234	135–233	25.78	6.53	chlo	40.04
30	CaHsp25.9	CA01g11490	1	690	1	229	128–228	25.93	8.36	chlo, mito	40.47
31	CaHsp26.5	CA10g10840	10	687	0	228	128–227	26.49	9.52	mito	50.30
32	CaHsp27.6	CA07g15940	7	756	5	251	156–250	27.63	8.99	chlo, nucl	54.52
33	CaHsp28.4	CA11g15400	11	765	1	254	30–109	28.35	8.35	golg, nucl, cyto	40.24
34	CaHsp30.1	Capana12g001708▴	12	804	1	267	35–127	30.14	8.26	Secretory pathway	47.98
35	CaHsp35.5	CA04g13680	4	942	1	313	35–129	35.53	7.70	cyto, nucl	33.82

*ORF, open reading frame; AA, amino acid; ACD, alpha-crystallin domain; Mol. Wt., molecular weight (kDa); pI, isoelectric point. Sequenced IDs marked with triangle are from Zunla-1 genome, with pentagram are from glabriusculum genome, others are from CM334 genome*.

The length of CaHsp20 proteins ranged from 141 (CaHsp16.4) to 313 amino acids (CaHsp35.5), and the predicted molecular weights of the CaHsp20s were between 16.33 kDa (CaHsp16.3) and 35.53 kDa (CaHsp35.5). The CaHsp20s shared a conserved ACD comprised about 90 amino acids, in which CaHsp24.3b owned the shortest ACD with 74 amino acids, while CaHsp16.3's ACD (116 amino acids) was the longest. The predicted pI-values of CaHsp20 proteins ranged from 5.07 (CaHsp18.4a) to 9.52 (CaHsp26.5), and the predicted instability indexes showed that only nine of the 35 CaHsp20s were stable proteins (instability index =40). In addition, it was also found that 16 (45.71%) of the total 35 *CaHsp20* genes had no introns, 18 genes (51.43%) had a single intron, while only *CaHsp27.6* had five introns.

### Characterization of the deduced amino acid sequences of CaHsp20s

**Figure 1:**
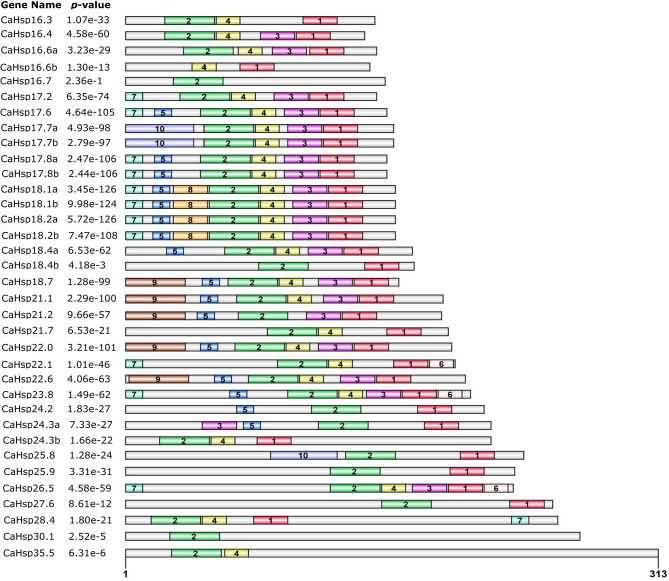
**Distribution of conserved motifs in the CaHsp20 family members**. Ten putative motifs are represented by a number in colored box. Names of all members and combined *p*-values are shown on the left side of the figure and motif sizes are indicated at the bottom of the figure. For details of motifs refer to Table 2.

**Figure 2:**
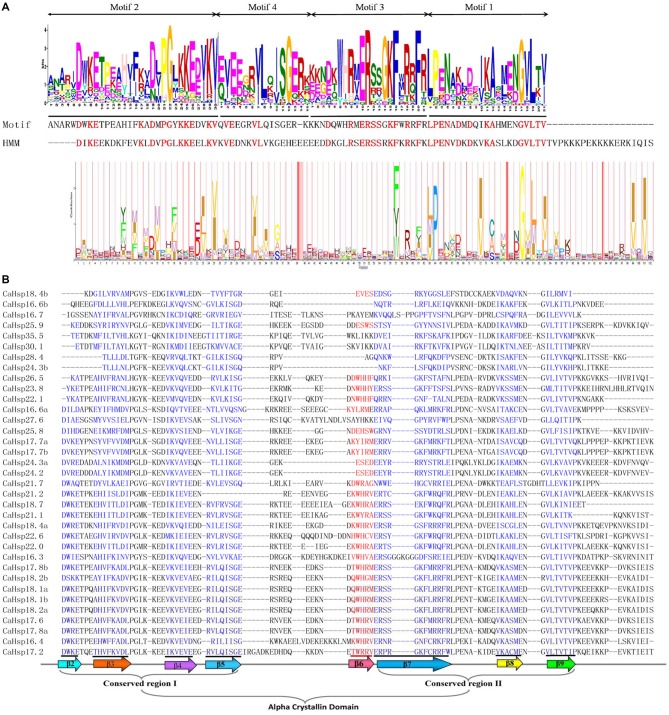
**Alignment the ACDs of CaHsp20s. (A)** Alignment of the ACD from the MEME results for the *CaHsp20* with Hsp20 from Pfam. The Motif 2, 4, 3, and 1 obtained via MEME analysis formed the putative CaHsp20 ACD, and the HMM logo from Pfam representing the Hsp20 domain (PF00011). The amino acids with red color indicate the matches between the MEME motifs and HMM sequences. **(B)** Alignment of the ACDs of *CaHsp20s* from pepper. Names of all members are shown on the left side of the figure. The alignment is performed by the Promals3D structural alignment program. Each predicted β-plated sheets are in blue. The primary structure of the ACD, including the conserved regions I (CRI), II (CRII), and β6-loop, is shown at the bottom of the figure.

Ten consensus motifs were found in CaHsp20 proteins by MEME motif search tool (Figure 1; Table 2). Based on the analysis of Pfam, SMART, and InterProScan, the full sequences of motif 1, 2, 3, and 4 formed the highly conserved ACD (Figure 2A), in which the combined sequence of motif 2 and 4 contained the CRI of ACD, while the combined sequence of motif 3 and 1 contained the β6-loop and CRII. The four motifs were detected in majority of the CaHsp20 proteins, while only motif 2 was found in CaHsp16.7 and CaHsp30.1, and CaHsp16.6b did not contain motif 2. Motif 6 was detected in CaHsp22.1, 23.8, and 26.5, and motif 8 was found behind the motif 5 in CaHsp18.1a, 18.1b, 18.2a, and 18.2b, while motif 10 were only found in the front of motif 2 in CaHsp17.7a, 17.7b, and 25.8 (Figure 1). Only motif 6 was found in the C-terminal regions, other motifs such as motif 5, 7, 8, 9, and 10 were found mainly in the N-terminal regions. It was worth noting that motif 9 (the signal-peptide sequence) was found in CaHsp18.7, 21.1, 21.2, 22.0, and 22.6, which was consistent with their prediction of secretory pathway localization (Table 1).

**Table 2:** **Motif sequences identified by MEME tools**.

**Motif**	**Length (aa)**	**Sequence**
1	21	LPENADMDQIKAHMENGVLTV
2	30	ANARWDWKETPEAHIFKADMPGYKKEDVKV
3	21	KKNDQWHRMERSSGKFWRRFR
4	15	QVEEGRVLQISGERK
5	11	DPFSMDLWDPF
6	15	TVPKEEVKKPDVKAI
7	11	MSLIPSFFGGR
8	21	GFPISSTIANTPSSARETSAF
9	36	MVKATVSLISFLVLAIVMAFLPSKNEALIPYTRPTW
10	41	MDFRLMGMDNTPIFHVLQHMMDTAGDESDKSVNAPSRNYVR

*Motif numbers corresponded to the motifs in Figure 1*.

The ACD was formed by two conserved regions, CRI (motif 2 and 4) with β2, β3, β4, and β5 and CRII (part of motif 3 and 1) with β7, β8, and β9, which were separated by a hydrophilic domain β6-loop (part of motif 3) with variable length. The multiple alignments of the conserved ACD domains, spanning ~90 amino acids in CaHsp20 proteins was clearly shown in Figure 2B. CaHsp16.6b, 16.7, 24.3b, 27.6, 28.4, 30.1, and 35.5 either lacked the β6-loop or contained sequences with unknown function between β5 and β7. The shorter ACDs of CaHsp18.4b, 24.3b, and 28.4 lacked the β2 (Figure 2B; Table 1), which might show the functional diversity and remains to be fully elucidated.

### Phylogenetic analysis of *CaHsp20* family

**Figure 3:**
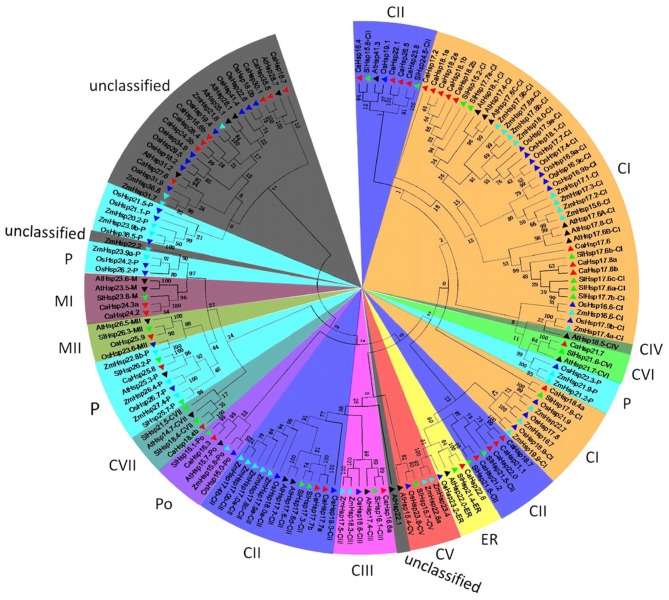
**Phylogenetic tree of pepper, tomato, and ***Arabidopsis*** Hsp20 proteins**. The Hsp20 proteins are used for construction of the phylogenetic tree using the MEGA 5.10 program. Unrooted Neighbor-Joining analysis is performed with pairwise deletion and Poisson correction. For the tomato (prefixed by *Sl*), *Arabidopsis* (prefixed by *At*), rice (prefixed by *Os*), and maize (prefixed by *Zm*) Hsp20 proteins, both gene names and subfamily names are used, and marked with green, black, blue, and cyan triangles, respectively. CaHsp20 proteins are marked with red triangles. The 12 subfamilies are marked by different colors, and the Hsp20 proteins unclassified have been colored in gray.

To analyze the evolutionary relationship of the *Hsp20* gene families in pepper, tomato, *Arabidopsis*, rice, and maize, the amino acid sequences of 154 Hsp20 proteins, including 35 from pepper, 24 from tomato, 25 from *Arabidopsis*, 35 from rice, and 35 from maize were aligned and used to construct an unrooted phylogenetic tree. As shown in Figure 3, 130 Hsp20s could be grouped into 12 distinct subfamilies, containing 47 CIs (cytosol/nucleus I), 27 CIIs, 6 CIIIs, 1 CIVs, 5 CVs, 3 CVIs, 4 CVIIs, 5 MIs (mitochondria I), 4 MIIs, 19 Ps (plastid), 4 ERs (endoplasmic reticulum), and 5 Pos (peroxisomal), respectively. However, the left 24 proteins from pepper, *Arabidopsis*, rice and maize were not well-clustered into any subfamily. The Hsp20 members from the five species were more closely to those in the same class in different species than to others in the same species. Many Hsp20s from the five species were clustered into class CI and CII, including 9 and 10 CaHsp20 proteins, respectively, which indicated that cytosol might be the main function area for CaHsp20s. The CI subfamily had two subclades that consisted of monocotyledon Hsp20s (OsHsp20s and ZmHsp20s) and two subclades that consisted of dicotyledon Hsp20s (CaHsp20s, SlHsp20s, and AtHsp20s), suggesting a single gene duplication took place prior to monocotyledon–dicotyledon bifurcation (Bondino et al., 2012). It was interesting that the members of P and M (MI and MII) subfamilies were closely related to each other, which was consistent with the Hsp20 of M subfamily evolved later from the P subfamily (Waters, 2013). It was noteworthy that no Hsp20 proteins from monocotyledons (rice and maize) were clustered into CIV cluster, which was consistent with the result suggested by Siddique et al. (2008) that CIV members of Hsp20 proteins were exclusively found in dicotyledonous. For the three species of dicotyledon, 12 pairs of orthologs (11 pairs of pepper/tomato orthologs and one pair of pepper/*Arabidopsis* otholog) were identified, indicating that the ancestral genes of Hsp20 family might have existed before differentiation of *Arabidopsis*, tomato and pepper species. In addition, several pairs of paralog were found in all of the three Hsp20 families from pepper, *Arabidopsis* and tomato, suggesting that *CaHsp20* genes underwent many rounds of duplication events occurred after the pepper speciation.

It was found that the phylogenetic classification of Hsp20 family was consistent with the pattern of intron type (pattern 1 had no intron, pattern 2 had one intron, and pattern 3 had more than one intron) in previous report (Ouyang et al., 2009). The most *CaHsp20* members of CI and ER subfamilies had no intron, and the members of CIII, CVII, P, and MII showed one intron (Figure 3; Table 1). However, six members belonged to CII subfamily showed no intron, other four members of CII subfamily had one intron. It was noteworthy that *CaHsp16.3* belonged to Po subfamily had one intron (pattern 2), which was different from the Po subfamily from rice (pattern 1; Ouyang et al., 2009). Among the unclassified *CaHsp20s, CaHsp27.6* was an orphan gene with five introns (pattern 3), which indicated particular phylogenetic status.

### Chromosomal localization and gene duplication of *CaHsp20* genes

**Figure 4:**
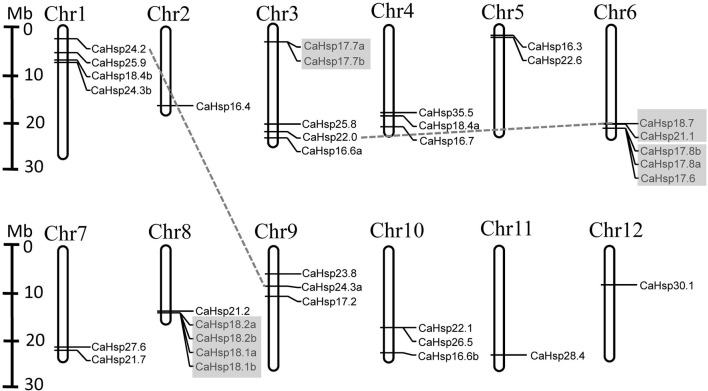
**Location and duplications of ***CaHsp20*** paralogs on pepper chromosomes**. The scale represents megabases (Mb). The chromosome numbers are indicated at the top of each bar. The *CaHsp20* genes present on duplicated chromosomal segments are connected by gray dotted lines and tandem duplicated genes are marked by gray rectangles.

The 35 *CaHsp20* genes were distributed on all 12 pepper chromosomes (Figure 4; Table 1). Among these 35 *CaHsp20* genes, five (14.3%) genes were located on each of chromosome 3, 6, and 8, four genes (11.4%) on chromosome 1, three genes on each of chromosome 4, 9, 10, two genes on each of chromosome 5 and 7, and only one gene on each of chromosome 2, 11, and 12. As gene duplication events involve in the evolution of plant genomes, the duplications of *CaHsp20s* were also analyzed. As shown in Figure 4, one segmental duplication event was detected between *CaHsp24.2* on chromosome 1 and *CaHsp24.3a* on chromosome 9, and three tandem duplication events (*CaHsp17.7a*/*17.7b, CaHsp17.6*/*17.8a*/*17.8b* and *CaHsp18.1a*/*18.1b*/*18.2a*/*18.2b*) were identified on chromosome 3, 6, and 8, respectively. However, *CaHsp18.7, 21.1*, and *22.0* were likely to originate from the same ancestor gene, which underwent one segmental duplication and one tandem duplication event during the pepper chromosome evolution. These results suggested that both tandem and segmental duplication events made an important contribution to pepper *CaHsp20* genes expansion.

### The interaction network of *CaHsp20* genes

**Figure 5:**
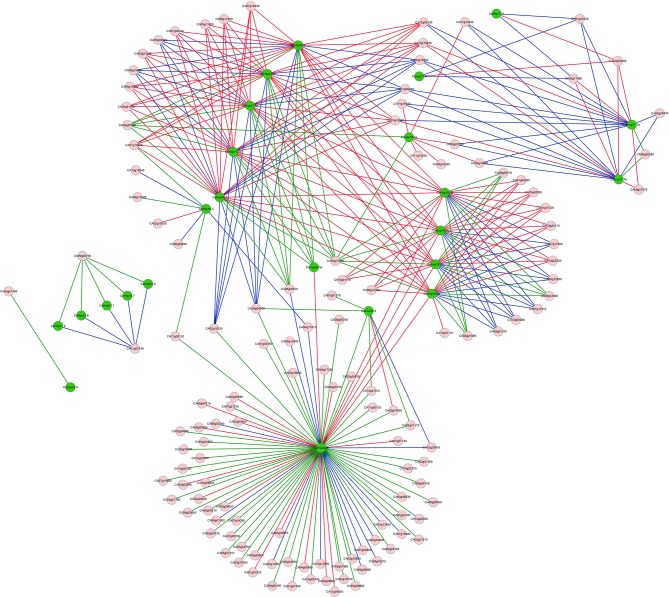
**The interaction network of ***CaHsp20*** genes in pepper according to the orthologs in ***Arabidopsis*****. The green circles represent the *CaHsp20* genes, and pink circles represent the pepper genes interaction with *CaHsp20* genes. The red lines represent the PCC > 0 (Pearson Correlation Coefficient), the blue lines mean the PCC < 0, and the green lines represent the PCC is not calculated.

To further analyze the relationship between *CaHsp20* genes and other genes, an interaction network of *CaHsp20* genes was built according to the orthologous to *Arabidopsis* (Figure 5). There were 24 (68.6%) out of 35 *CaHsp20* genes orthologously related to 13 (68.4%) out of 19 *Arabidopsis Hsp20* genes. The red, blue, and green lines stand for Pearson correlation coefficients >0, < 0, and not calculated, which were corresponding to positive correlation (136 pairs of interacting genes), negative correlation (99 pairs), and unclear correlation (106 pairs), respectively. The interaction network of *CaHsp20* genes showed a complex functional relationship. For example *CaHsp24.2* directly or indirectly interacted with many other *CaHsp20* genes (*CaHsp17.8a, 17.8b, 22.1, 23.8, 26.5, 18.1a, 18.1b, 18.2a*, and *18.2b*), and also had more interacted genes than other *CaHsp20s*, while only one gene (*CA03g01490*) interacted with *CaHsp25.8*. In addition, *CaHsp18.1a, 18.1b, 18.2a*, and *18.2b* genes interacted with heat stress related genes (heat shock protein 70 family genes *CA03g30260* and *CA01g31330*), *CaHsp17.7a* and *17.7b* genes interacted with heavy metal stress related gene (heavy metal transport/detoxification superfamily protein gene *CA02g24070*), *CaHsp16.3* interacted with signal transduction related gene (calcium-binding EF-hand family protein gene *CA10g18540*), and *CaHsp25.9* with transcription factor (MADS-box transcription factor family protein gene *CA00g74550*), which might indicate that *CaHsp20* genes involved in many response mechanisms by regulating their downstream genes or being regulated by their upstream regulators in pepper.

### Expression patterns of *CaHsp20* genes in different tissues

**Figure 6:**
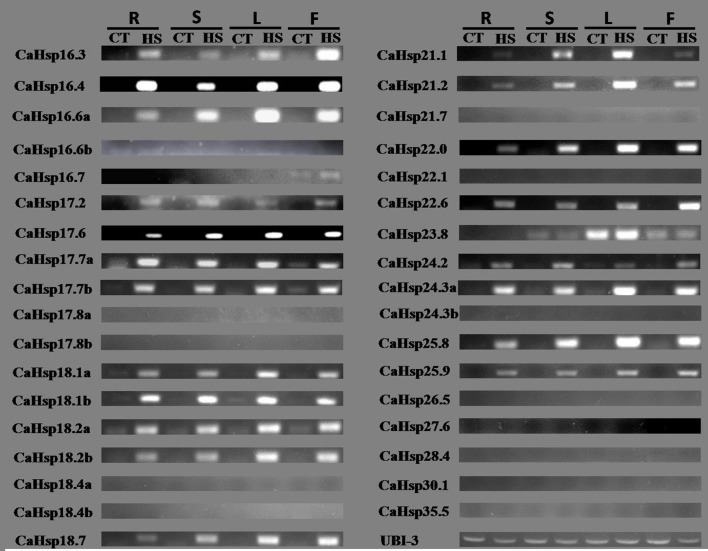
**RT-PCR analysis of expression pattern of 35 ***CaHsp20*** genes in four pepper tissues under normal and HS conditions**. R, root; S, stem; L, leaf; F, flower. Normal condition: 26°C for 2 h; HS condition: 40°C for 2 h. The pepper ubiquitin-conjugating protein gene (*CaUBI-3*) is used as an internal control.

To primarily investigate the biological functions of individual *CaHsp20* members in pepper, the heat-induced expression of these *CaHsp20* genes were examined by semi-quantitative RT-PCR in four major tissues (root, stem, leaf, and flower) from thermotolerant line R9 (Figure 6). Under normal temperature condition, 27 of the 35 *CaHsp20* genes showed no significant tissue specificity as they were barely expressed in the tested tissues, however, the left eight *CaHsp20* genes, including *CaHsp16.3, 16.6b, 16.7, 17.7a, 17.7b, 18.1b, 18.2a*, and *23.8* could be detected at least in one tissue. Twenty-two (62.9%) *CaHsp20* genes were highly induced in at least one tissue after HS treatment (40°C for 2 h). For example *CaHsp16.3, 16.4, 17.6*, and *18.1b* were expressed at relatively high levels in all of tissues, while *CaHsp16.7* and *23.8* were highly induced by HS only in flower or leaf, respectively. Many heat-induced *CaHsp20s*, such as *CaHsp16.6a, 17.6, 21.1, 21.2, 22.0, 24.3a*, and *25.8* were expressed at higher level in treated pepper leaves than other three tissues, however, the highest levels of *CaHsp16.3* and *22.6* were detected in flowers with HS treatment, and the expression level of *CaHsp17.7a* was highest in treated roots. The transcripts of 12 (34.3%) genes (*CaHsp17.8a, 17.8b, 18.4a, 18.4b, 21.7, 22.1, 24.3b, 26.5, 27.6, 28.4, 30.1*, and *35.5*) were not visible in the both of the normal temperature and HS conditions.

### Expression profiles of *CaHsp20* genes in response to heat stress

**Figure 7:**
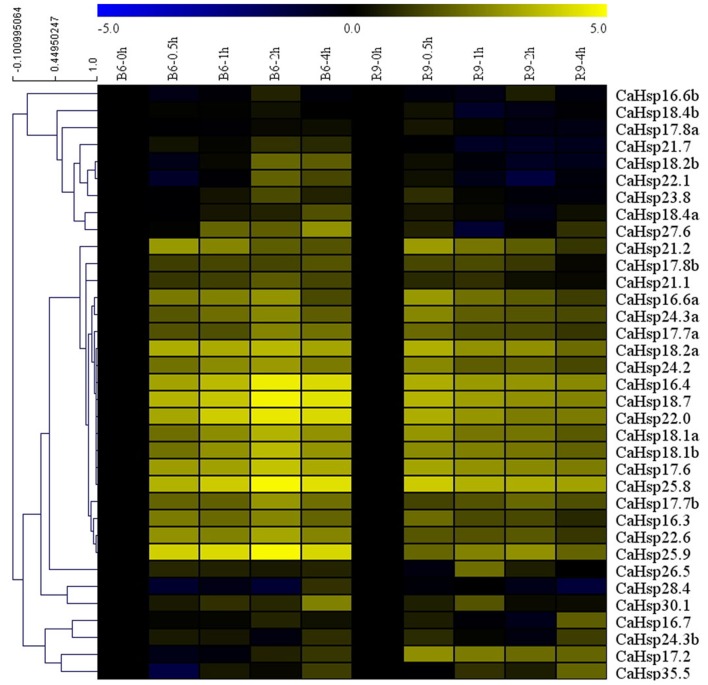
**Heat map showing ***CaHsp20*** genes expression pattern in pepper thermosensitive line B6 and thermotolerant line R9 under HS condition**. Leaves of B6 and R9 seedlings (6–8 true leaves) are used to test the changes of *CaHsp20* genes expression level at different time points (0, 0.5, 1, 2, and 4 h) with HS treatment (40°C). *CaUBI-3* is used as an internal control. qRT-PCR data are shown relative to 0 h. The relative expression levels were calculated using the 2^−ΔΔCT^ method, and normalized using log10. The heat map was created using MeV.

In order to investigate the heat response profile for *CaHsp20* genes, the transcript patterns of each *CaHsp20* in leaves from thermosensitive line B6 and thermotolerant line R9 with continuous heat stress treatment (40°C for 4 h) were compared. As shown in Figure 7, strong response to HS in all of the 35 *CaHsp20* genes from both lines (B6 and R9) were observed, in which a majority of these genes were up-regulated by HS treatment. For example *CaHsp16.4, 18.7, 22.0, 25.8*, and *25.9* were highly induced after a short-term HS (40°C for 0.5 h) and their high expression levels were maintained until the end of the HS treatment, while only *CaHsp21.7* and *28.4* were down-regulated by HS in R9. Generally, the peaks of relative expression levels of *CaHsp20* genes (compared with HS for 0 h) in thermosensitive line B6 treated with HS were higher than those of thermotolerant line R9, however the appearance of peak values were observed at 2 or 4 h in B6 but at 0.5 h in R9 after HS treatment. In addition, it was worth noting that compared with other *CaHsp20* members, *CaHsp16.4, 18.7, 22.0, 25.8*, and *25.9* which were significantly up-regulated in B6 leaves treated with HS, however, no main heat-response genes was detected in line R9 after HS treatment, although many *CaHsp20* genes were induced by HS in R9.

### Analysis of stress-related *cis*-elements in the *CaHsp20* promoters

**Figure 8:**
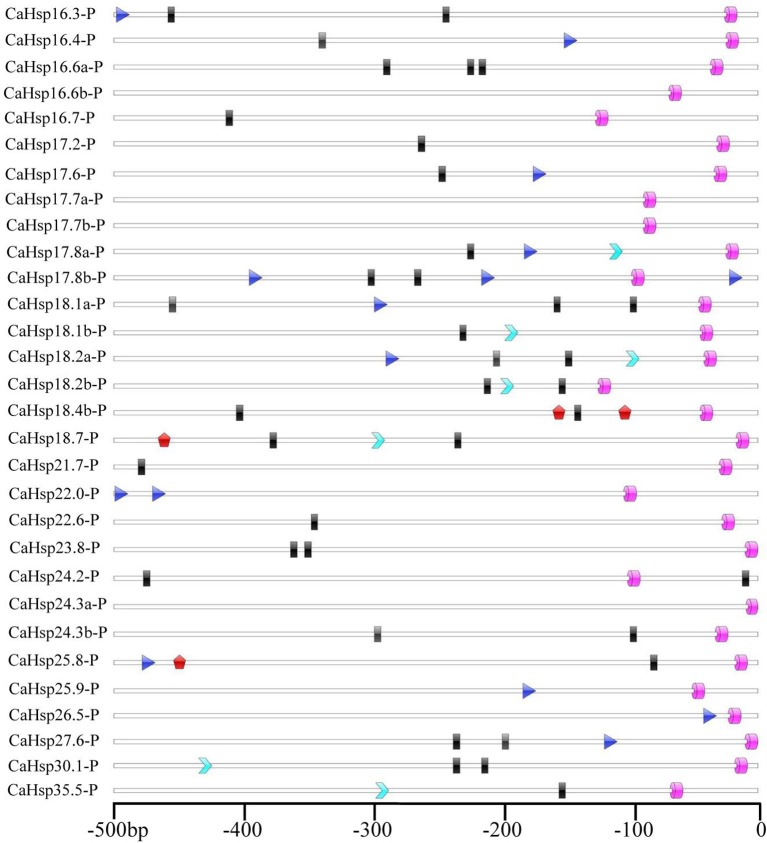
**Predicted ***cis***-elements in the promoter regions of ***CaHsp20*** genes**. Promoter sequences (–500 bp) for 30 *CaHsp20* genes (promoter regions of *CaHsp18.4a, 21.1, 21.2, 22.1*, and *28.4* were absent) are analyzed. The names of the promoters of *CaHsp20* genes are shown on the left side of the figure. The number at the bottom indicates the number of nucleotides upstream to the translation initiation codon, ATG. The blue triangles stand for the heat shock elements (HSEs), the pink cylinders for the TATA-box elements, the sky blue arrows for the TC-rich repeats, the black rectangles for the CCAAT elements, and the red pentagons for the W-box elements.

To further explore the possible regulation mechanism of *CaHsp20* genes in the HS response of pepper, the *cis*-elements involving in the activation of defense-related genes in the promoter regions of *CaHsp20s* were also scanned, and the promoter regions (–500 bp upstream of the translation start site) of a total of 30 *CaHsp20* genes (promoter regions of *CaHsp18.4a, 21.1, 21.2, 22.1*, and *28.4* were absent) from PGD were used (Figure 8). One to three HSE elements (heat stress responsiveness) were found in the promoter regions of 12 genes (*CaHsp16.3, 16.4, 17.6, 17.8a, 17.8b, 18.1a, 18.2a, 22.0, 25.8, 25.9, 26.5*, and *27.6*), respectively, in which the number of HSE elements in the promoter region of *CaHsp17.8b* was the maximum. Although HSE elements could not be found in −500 bp promoter regions of other 11 *CaHsp20* genes (*CaHsp16.7, 17.2, 17.7b, 18.1b, 18.4b, 21.7, 23.8, 24.2, 24.3b, 30.1*, and *35.5*), 1–3 HSE elements were detected in their −1500 bp promoter regions, however, no any HSE element was present in either −1500 or −500 bp promoter regions of the rest seven *CaHsp20s* (*CaHsp16.6a, 16.6b, 17.7a, 18.2b, 18.7, 22.6*, and *24.3a*). In addition, other stress-related *cis*-elements were detected, including TATA-boxes in all 30 selected promoter regions, CCAAT-motif in 23 genes, TC-rich repeats in seven genes (*CaHsp17.8a, 18.1b, 18.2a, 18.2b, 18.7, 30.1*, and *35.5*), and W-box in three genes (*CaHsp18.4b, 18.7*, and *25.8*).

## Discussion

It has been demonstrated that Hsp20s can prevent the irreversible aggregation of denaturing proteins and enhance plant-acquired thermotolerance (Wang et al., 2004; Haslbeck and Vierling, 2015). Benefiting from the availability of whole genome sequence in recent years, several Hsp20 families have been identified from *Arabidopsis*, rice, soybean, and *Populus trchocarpa* (Waters et al., 2008; Lopes-Caitar et al., 2013), however, very little is known about this family in pepper.

The present study describes the identification of a total of 35 *CaHsp20* genes from pepper, investigation of their structure, chromosomal organization, evolutionary relationships, prediction of interaction network, and expression patterns across the different tissues and under heat stress condition, which provides a basis for comprehensively understanding the *CaHsp20* gene family in pepper and makes a base for future functional divergence of the *Hsp20* genes in the Solanaceae family.

Although having the most diversity among the molecular chaperone families, the Hsp20s share a conserved structural domain, the ACD flanked by a non-conserved N-terminal sequence of variable length and a conserved short C-terminal sequence (Kriehuber et al., 2010; Bondino et al., 2012). Characterization and multiple sequence alignments divulged that all selected 35 CaHsp20s contained the ACD with various sequences. The CRI (motif 2 and 4) consisted of β2, β3, β4, and β5 and CRII (part of motif 3 and 1) consisted of β7, β8, and β9 in ACD were structurally conserved, containing the signature typical residues Pro-X(14)-Gly-Val-Leu and Pro-X(14)-X-Val/Leu/Ile-Val/Leu/Ile, respectively (Figures 1, 2B; Caspers et al., 1995; Ouyang et al., 2009). Most of the CaHsp20 ACDs also contain the β6 sheet, the non-conserved motif connecting β5 in CRI and β7 in CRII (Bondino et al., 2012), while it is replaced by sequences of unknown function in four *CaHsp20* members (*CaHsp16.7, 27.6, 30.1*, and *35.5*) or absent in three members (*CaHsp16.6b, 24.3b*, and *28.4*). Plant Hsp20 proteins of CIV clusters lack the β6 sheet (Waters, 2013) but more interesting is that none of the Hsp20 members from pepper, tomato, rice, and maize are found in the CIV cluster, while found in *Arabidopsis* Hsp20 family, in addition, the above seven *CaHsp20* genes are not clustered into any subfamily (Figure 3), it can be inferred that these genes from pepper belong to CIV cluster or form a new cluster. Because the β6 sheet is crucial for dimer formation and oliogomerization, whether these CaHsp20 genes lacking of β6 sheet can function as a molecular chaperone still need further comparative studies.

Gene organization also plays an important role in the evolution of multiple gene families and in response to stress conditions (Xu et al., 2012). Sixteen *CaHsp20* genes are intronless (pattern 1), 18 genes contain one intron (pattern 2), only one gene (*CaHsp27.6*) contains five introns (pattern 3; Table 1). Most *CaHsp20* members of CI and ER subfamilies have no intron (pattern 1) and the members of MII subfamily have one intron (pattern 2; Figure 3; Table 1), which is consist with rice *Hsp20* family, while the patterns of *CaHsp20* members and *Hsp20s* from rice belong to CII, Po and P subfamilies are not the same (Ouyang et al., 2009), indicating that although intron patterns shed light on the phylogenetic relationship, there is difference between different species. Most of these *CaHsp20* genes are highly induced by HS in different tissues (Figure 6), which may indicate that the expression levels of genes without introns or with a short length intron will be enhanced under HS in plants (Lopes-Caitar et al., 2013). The compact genes have faster response to HS and protect plants from HS damage better than those genes contain many introns, and the compact genes are gradually selected by stress conditions during the long-term evolution (Jeffares et al., 2008), which may be the reason that *Hsp20* family members form a first line of defense against protein aggregation caused by HS condition in order to maintain protein homeostasis and enhance the plant thermotolerance (Haslbeck and Vierling, 2015).

Gene duplication events play a significant role in expansions of gene family members and evolutionary mechanisms of genomes (Vision et al., 2000). Tandem duplications and segmental duplications are the major evolutionary patterns (Kong et al., 2007). In this analysis, 24 *Hsp20* genes were identified in tomato, however, in pepper with a genome size four-fold larger than that of tomato, only 35 *CaHsp20* genes were found. The reason for this might be the whole-genome duplication (WGD) events in pepper and tomato (Qin et al., 2014). A total of 14 *CaHsp20* duplicated genes were found in pepper chromosomes, including one segmental duplication event (*CaHsp24.2*/*CaHsp24.3a*) and three tandem duplication events (*CaHsp17.7a*/*17.7b, CaHsp17.6*/*17.8a*/*17.8b* and *CaHsp18.1a*/*18.1b*/*18.2a*/*18.2b*; Figure 4), contributing to evolution of MI, CI and CII genes. Besides, *CaHsp18.7, 21.1*, and 22.0 experienced one segmental duplication and one tandem duplication event, also contributing to evolution of CII genes. In previous report, the CI subfamily in plant including tomato, is always the largest (Waters, 2013), however the number of *CaHsp20* members belong to CII subfamily is largest (Figure 3), which is not consistent with that of the other plants. The reason may be attributed to *CaHsp20* genes of CII subfamily appeared after speciation through multiple gene duplication. Due to the opinion that gene duplication in tandem mainly occurs in the genes encoded membrane proteins and stress-related proteins, we can suggest that the dominant tandem duplications in *CaHsp20* genes may be related to the fact that *CaHsp20s* are regulated by HS (Cannon et al., 2004; Rizzon et al., 2006; Hanada et al., 2008; Giorno et al., 2012).

The tissue-specific expression patterns of *Hsp20* genes have been reported in various species, such as *Arabidopsis*, rice, and soybean, however, there is no uniform pattern of gene expression for all the plant *Hsp20* genes (Lopes-Caitar et al., 2013; Waters, 2013). Most *CaHsp20* genes have no or low expression in the four examined tissues (root, stem, leaf, and flower) from pepper thermotolerant line R9 under normal temperature condition, but are strongly induced by HS (Figure 6), which is largely consistent with the previous reported that almost all the plant *sHsps* are heat induced. Among the 35 *CaHsp20* genes, *CaHsp23.8* possesses the most distinctive expression pattern compared with other *CaHsp20* genes, which expresses constitutively in stems, leaves and flowers, but almost no induction by HS. Waters (2013) reported that *AtHsp21.7CV* gene lacking chaperone activity from *Arabidopsis* is constitutively expressed in shoots and not up-regulated by HS, therefore, it can be assumed that *CaHsp23.8* also may lack the chaperone activity, but it needs more scientific evidence. In addition, the expression of *CaHsp16.6b, 16.7, 17.7a, 17.7b*, and *18.2a* were detected in various tissues under normal condition, we can suggest that these genes exhibit specific housekeeping activities as with some *Hsp20* genes from rice and soybean (Ouyang et al., 2009; Lopes-Caitar et al., 2013).

It has been demonstrated that sHsps are associated with thermotolerance in various plant species, however, many Hsp20s exist in plants which may lead to function redundancy of individual genes (Sun and MacRae, 2005; Waters, 2013). The comprehensive analysis of the expression for individual *Hsp20* members under HS is necessary for further in-depth functional analyses of *Hsp20* genes in plant thermotolerance. In this study, the dynamic expression of all 35 *CaHsp20s* in thermosensitive line B6 and thermotolerant line R9 with HS treatment were analyzed (Figure 7). Almost all *CaHsp20* genes are induced by HS treatment in line both B6 and R9, indicating that the accumulation of the CaHsp20s effectively reduce the damage from HS and enhance pepper thermotolerance by binding denaturing proteins and preventing them from irreversible aggregation (Haslbeck and Vierling, 2015). It is well-known that *Hsfs* are activated under HS condition, and then bind the HSE elements of the promoters of the *Hsp* genes to regulate the expression of downstream *Hsp* genes. The results that the promoter regions of *CaHsp20* genes contain various *cis*-elements (Figure 8) and *CaHsp20s* can interact with many functional genes (Figure 5) suggest that the expression regulation network of *CaHsp20* genes under HS condition is a complex signal transduction pathway, not just *Hsfs*. This hypothesis can be supported by the observation that some *CaHsp20* genes (*CaHsp16.6a, 17.7a, 18.7, 21.2, 22.6*, and *24.3*) whose promoter regions (–1500 bp) do not contain the HSEs can be highly induced by HS treatment. Intriguingly, *CaHsp17.7a* and *17.7b* genes are in a tandem duplication share a similar expression pattern in both lines B6 and R9 with HS treatment, however, another tandem duplication pair *CaHsp18.2a* and *18.2b* exhibit different expression patterns in both lines B6 and R9, so it seems that not all the members of the same *sHsp* subfamily have the same expression patterns (Waters, 2013). It is worth noting that the peak of the expression of most *CaHsp20* genes in R9 (HS for 0.5 or 1 h) appeared earlier than in B6 (HS for 2 or 4 h), which may indicate that when suffering from HS, the pepper thermotolerant line R9 can start the thermal response mechanism faster than the thermosensitive line B6, however, the relationship between the function of *CaHsp20* genes and thermotolerant mechanism in pepper need to be further studied.

## Conclusions

In this study, a genome-wide analysis of *CaHsp20* gene family in pepper was performed to reveal gene structure, phylogenetic relationship, evolution, and expression profiles in different tissues (root, stem, leaf, and flower) and HS conditions. Most *CaHsp20* genes maintained the low expression level under normal temperature condition and could be highly induced by HS, indicating that they played a key role in the process of pepper acquired thermotolerance. The data in this study will provide a foundation for functional characterization of *CaHsp20* family members, and make a contribution toward illuminating the mechanism of pepper acquired thermotolerance, and even the adaptability of pepper to other stress conditions.

### Conflict of interest statement

The authors declare that the research was conducted in the absence of any commercial or financial relationships that could be construed as a potential conflict of interest.

## Supplementary material

Figure S1 **The logic flowchart of identification of pepper ***Hsp20*** genes**.

Table S1 **The gene IDs of ***Hsp20*** members from ***Arabidopsis***, tomato, maize and rice**.

Table S2 **Primer sequences used for semi-quantitative RT-PCR and quantitative real-time PCR analysis**.
