# Supplementary figures and images for: Genome-wide analysis of the CaHsp20 gene family in pepper: comprehensive sequence and expression profile analysis under heat stress
Source: Front Plant Sci. 2015 Oct 1;6:806. doi: 10.3389/fpls.2015.00806 (PMC4589653; doi:10.3389/fpls.2015.00806)

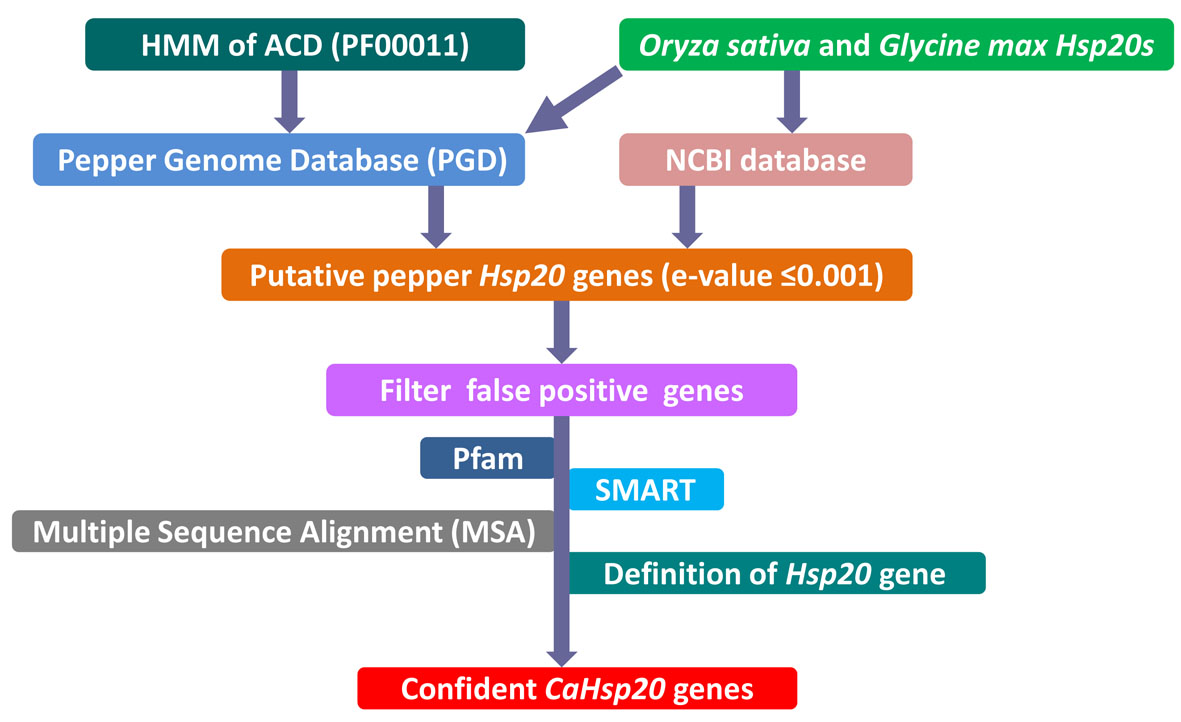

Supplement: Figure S1 — The logic flowchart of identification of pepper Hsp20 genes. [file Image1.JPEG]
