# Supplementary material for: Genome-wide analysis of the CaHsp20 gene family in pepper: comprehensive sequence and expression profile analysis under heat stress
Source: Front Plant Sci. 2015 Oct 1;6:806. doi: 10.3389/fpls.2015.00806 (PMC4589653; doi:10.3389/fpls.2015.00806)
Supplement: Table S1 — The gene IDs of Hsp20 members from Arabidopsis, tomato, maize and rice. [file Table1.DOCX]

**Table S1 The gene IDs of *Hsp20* members from *Arabidopsis*, tomato, maize and rice.**

| ***Arabidopsis thaliana*** | | | ***Solanum lycopersicum*** | | | ***Zea mays*** | | | ***Oryza sativa*** | |
| --- | --- | --- | --- | --- | --- | --- | --- | --- | --- | --- |
| **Gene name** | | **Gene ID** | **Gene name** | | **Gene ID** | **Gene name** | | **Gene ID** | **Gene name** | **Gene ID** |
| AtHsp17.4-CI | At3g46230 | | SlHsp15.2-CI | Solyc09g015000 | | ZmHsp17.2-CI | GRMZM2G158232_T01 | | OsHsp23.6-MII | LOC_Os02g10710 |
| AtHsp17.6C-CI | At1g53540 | | SlHsp17.7a-CI | Solyc09g015020 | | ZmHsp17.8a-CI | AC208204.3_FGT006 | | OsHsp16.9a-CI | LOC_Os01g04380 |
| AtHsp18.1-CI | At5g59720 | | SlHsp17.9-CI | Solyc04g072250 | | ZmHsp31.7 | GRMZM2G081822_T01 | | OsHsp17.9a-CI | LOC_Os03g15960 |
| AtHsp17.6A-CI | At1g59860 | | SlHsp17.6a-CI | Solyc06g076560 | | ZmHsp16.6-CI | GRMZM2G049767_T01 | | OsHsp18.9-CI | LOC_Os02g03570 |
| AtHsp17.8-CI | At1g07400 | | SlHsp17.7b-CI | Solyc06g076520 | | ZmHsp15.8-Po | GRMZM2G335242_T01 | | OsHsp34.9 | LOC_Os05g51440 |
| AtHsp17.6B-CI | At2g29500 | | SlHsp17.6b-CI | Solyc06g076570 | | ZmHsp36.8 | GRMZM2G037146_T01 | | OsHsp24.2-P | LOC_Os02g52150 |
| AtHsp17.6-CII | At5g12020 | | SlHsp17.6c-CI | Solyc06g076540 | | ZmHsp17.1-CI | GRMZM2G437100_T01 | | OsHsp17.9b-CI | LOC_Os01g04350 |
| AtHsp17.7-CII | At5g12030 | | SlHsp17.3-CII | Solyc08g062340 | | ZmHsp19.9-CI | GRMZM2G324956_T01 | | OsHsp18.0a-CII | LOC_Os01g08860 |
| AtHsp17.4-CIII | At1g54050 | | SlHsp17.6d-CII | Solyc08g062450 | | ZmHsp22.7 | GRMZM2G375517_T01 | | OsHsp16.6-CI | LOC_Os01g04340 |
| AtHsp15.4-CV | At4g21870 | | SlHsp21.5-CII | Solyc03g113930 | | ZmHsp17.0a-CII | GRMZM2G404249_T01 | | OsHsp21.9 | LOC_Os11g13980 |
| AtHsp21.7-CVI | At5g54660 | | SlHsp21.6-CII | Solyc01g102960 | | ZmHsp17.0b-CII | GRMZM5G899188_T01 | | OsHsp21.1-P | LOC_Os10g07210 |
| AtHsp14.7-CVII | At5g47600 | | SlHsp24.5-CII | Solyc09g011710 | | ZmHsp17.8b-CI | GRMZM2G046382_T01 | | OsHsp19.0-CII | LOC_Os02g12610 |
| AtHsp23.5-M | At5g51440 | | SlHsp15.6-CII | Solyc02g093600 | | ZmHsp17.9a-CII | GRMZM2G083810_T01 | | OsHsp38.5-P | LOC_Os10g07200 |
| AtHsp23.6-M | At4g25200 | | SlHsp16.1-CIII | Solyc03g123540 | | ZmHsp18.3-CIII | GRMZM2G098167_T01 | | OsHsp19.4 | LOC_Os10g30180 |
| AtHsp26.5-MII | At1g52560 | | SlHsp15.7-CV | Solyc02g080410 | | ZmHsp17.4a-CI | GRMZM2G413897_T01 | | OsHsp16.9b-CI | LOC_Os01g04360 |
| AtHsp22.0-ER | At4g10250 | | SlHsp21.6-CVI | Solyc07g064020 | | ZmHsp17.4b-CII | GRMZM2G012455_T01 | | OsHsp17.7-CI | LOC_Os03g16040 |
| AtHsp15.7-Po | At5g37670 | | SlHsp18.4-CVII | Solyc08g078720 | | ZmHsp17.5-CIII | GRMZM2G085934_T01 | | OsHsp26.2-P | LOC_Os06g11610 |
| AtHsp25.3-P | At4g27670 | | SlHsp21.5-CVII | Solyc08g078710 | | ZmHsp23.9a-P | GRMZM2G007729_T01 | | OsHsp23.2-ER | LOC_Os04g36750 |
| AtHsp18.5-CIV | At2g19310 | | SlHsp23.8-M | Solyc08g078700 | | ZmHsp22.8a | GRMZM2G331701_T01 | | OsHsp18.6-CIII | LOC_Os02g54140 |
| AtHsp22.1 | At3g22530 | | SlHsp26.3-MII | Solyc12g042830 | | ZmHsp21.9-P | GRMZM2G013970_T01 | | OsHsp21.5-P | LOC_Os09g17660 |
| AtHsp25.1 | At2g27140 | | SlHsp21.4-ER | Solyc11g020330 | | ZmHsp16.7 | GRMZM2G481605_T01 | | OsHsp23.8-CV | LOC_Os07g33350 |
| AtHsp28.1 | At5g20970 | | SlHsp16.1-Po | Solyc04g014480 | | ZmHsp17.8c-CII | GRMZM2G034157_T01 | | OsHsp17.4-CI | LOC_Os03g16020 |
| AtHsp28.7 | At1g76770 | | SlHsp26.2-P | Solyc03g082420 | | ZmHsp20.2-P | GRMZM2G109814_T01 | | OsHsp18.1-CI | LOC_Os03g16030 |
| AtHsp31.2 | At1g06460 | | SlHsp25.7-P | Solyc05g014280 | | ZmHsp21.2-P | GRMZM2G010927_T01 | | OsHsp16.0-Po | LOC_Os06g14240 |
| AtHsp41.3 | At5g04890 | |  |  | | ZmHsp17.9b-CI | GRMZM2G306679_T01 | | OsHsp41.4 | LOC_Os03g45330 |
|  |  | |  |  | | ZmHsp15.6-CI | GRMZM2G422240_T01 | | OsHsp31.9 | LOC_Os03g45340 |
|  |  | |  |  | | ZmHsp22.2 | GRMZM2G135960_T01 | | OsHsp26.7-P | LOC_Os03g14180 |
|  |  | |  |  | | ZmHsp22.8b-P | GRMZM5G803365_T01 | | OsHsp22.3-P | LOC_Os05g42120 |
|  |  | |  |  | | ZmHsp26.4-P | GRMZM2G149647_T01 | | OsHsp19.1 | LOC_Os03g61940 |
|  |  | |  |  | | ZmHsp23.9b-P | AC210517.3_FGT003 | | OsHsp22.6 | LOC_Os03g06170 |
|  |  | |  |  | | ZmHsp23.4 | GRMZM2G346839_T01 | | OsHsp17.8 | LOC_Os02g48140 |
|  |  | |  |  | | ZmHsp27.4-P | GRMZM2G080724_T01 | | OsHsp18.2 | LOC_Os01g40530 |
|  |  | |  |  | | ZmHsp18.0-CI | GRMZM2G479260_T01 | | OsHsp16.9c-CI | LOC_Os01g04370 |
|  |  | |  |  | | ZmHsp21.6 | GRMZM2G465723_T01 | | OsHsp18.0b | LOC_Os10g30162 |
|  |  | |  |  | | ZmHsp17.3-CI | GRMZM2G333635_T01 | | OsHsp29.5 | LOC_Os01g40550 |
