# Supplementary material for: Genome-wide analysis of the CaHsp20 gene family in pepper: comprehensive sequence and expression profile analysis under heat stress
Source: Front Plant Sci. 2015 Oct 1;6:806. doi: 10.3389/fpls.2015.00806 (PMC4589653; doi:10.3389/fpls.2015.00806)
Supplement: Table S2 — Primer sequences used for semi-quantitative RT-PCR and quantitative real-time PCR analysis. [file Table2.DOCX]

**Table S2 The primer sequences of 35 *CaHsp20* genes.**

| **Gene** | **Forward Primer** | **Reverse Primer** |
| --- | --- | --- |
| qCaHsp16.3 | 5' GTCTATTTTTGGTGATCCATTTAG 3' | 5' GAAGAGCATTAGAACCATATGATG 3' |
| qCaHsp16.4 | 5' ATGTCAAAGATGATCAGCTTATTG 3' | 5' GGGTACTCAACACGGGACAC 3' |
| qCaHsp16.6a | 5' GATGTAGTGAACCATCTTCTCAAC 3' | 5' GGAATGTTACCCTTGTTCTCAC 3' |
| qCaHsp16.6b | 5' TAACAACAACATCAAATAAATCAAG 3' | 5' AACTACAACCACGATAGCTGC 3' |
| qCaHsp16.7 | 5' AGATGGCATTCTGGAAGTCGT 3' | 5' TCAAGAAACTGGGAAAGAGGC 3' |
| qCaHsp17.2 | 5' TGCTCGACGATATTTTCCAAC 3' | 5' GGCATATTTAGAGGTGGTTTCAT 3' |
| qCaHsp17.6 | 5' GATCCCAAGAATCTTCGGC 3' | 5' AGCCTAATTCCCTGAACGG 3' |
| qCaHsp17.7a | 5' GGCTGATGGGTATGGACAAC 3' | 5' TGGGGCATTCACAGACTTG 3' |
| qCaHsp17.7b | 5' GGATTTCAGGCTGATGGGTAT 3' | 5' CGGGGCATTCACAGACTTG 3' |
| qCaHsp17.8a | 5' CCCTAAGCAGGTATAAATACCTACC 3' | 5' GAGAATTATTTGTATTTGCTTGGC 3' |
| qCaHsp17.8b | 5' AATACCCACCAGCTCCTCTT 3' | 5' GCTTGAATTTTCTTTGTTTGC 3' |
| qCaHsp18.1a | 5' TGGTCGCAGGAGCAACAT 3' | 5' GTGTTGGCGATTGTGCTTG 3' |
| qCaHsp18.1b | 5' GGTCGCAGGAGCAACATCT 3' | 5' GTGTTGGCGATTGTGCTTG 3' |
| qCaHsp18.2a | 5' CAGCAAATCAAACACTACTATCAAAG 3' | 5' TGGGGAATTTGTTGTTTGAAG 3' |
| qCaHsp18.2b | 5' CAACAACAAATTATCAAACGCTAC 3' | 5' GTTGTTTGAAGAAGACAAAAGATGT 3' |
| qCaHsp18.4a | 5' TGGCAGTCCATTTTCTTCAG 3' | 5' CATCATCCCCACCAGCAC 3' |
| qCaHsp18.4b | 5' TTCTTCTACTAGCACCACAGATTC 3' | 5' GTATCAACTTCCAGCACTTCCT 3' |
| qCaHsp18.7 | 5' TCCATCAAAAAATGAGGCACT 3' | 5' ATGGTGAGTGGGGTTTGTTC 3' |
| qCaHsp21.1 | 5' TATTGCTATGGCCTTTCTTCC 3' | 5' AATTGATTCTACACCTTTTGGG 3' |
| qCaHsp21.2 | 5' AATCACCTTATTAGCCCGTTC 3' | 5' ATGCCATTTCTCGCCTTC 3' |
| qCaHsp21.7 | 5' GGTGTGTTCCATTAAAAGAAGATG 3' | 5' AACCTTCACACAAAGCCTTATGT 3' |
| qCaHsp22.0 | 5' CCATCAAAAACTGAGGCACTG 3' | 5' ATGGTGAGTGGGGTTTGTTC 3' |
| qCaHsp22.1 | 5' TTAGTAGCTCTATTTAACGGTGGC 3' | 5' TTCTGATTTGTTTGGAATTGATGT 3' |
| qCaHsp22.6 | 5' TGCAGGTGGCATCAATAGG 3' | 5' AATCGAAAGACTCTCCTCCTTG 3' |
| qCaHsp23.8 | 5' GCTCATGAAGTTTGGGATTCTT 3' | 5' ATGGTGCTGGGGCTATGAG 3' |
| qCaHsp24.2 | 5' CTTGCTCTTAGGAGGGCTACC 3' | 5' TCTGAGTGTTAGTATTGAAGGATCG 3' |
| qCaHsp24.3a | 5' GGTTTACCCAGTTTCTTCTCAG 3' | 5' GGTCCATCATGTTTAGCAGC 3' |
| qCaHsp24.3b | 5' AAGATGACACAACACGAAATGC 3' | 5' AACATAACGACCGAAACCAAG 3' |
| qCaHsp25.8 | 5' CTTGTTCTGTTTTCTTTCCGTC 3' | 5' GATGTGTCTTTGTTATCTCCAGTG 3' |
| qCaHsp25.9 | 5' GTTAGCCAAGTGAAAAATGTAGAG 3' | 5' TTGCCACTTTCAGAGACAGC 3' |
| qCaHsp26.5 | 5' GCCACGACAGTTACGCATC 3' | 5' GGGTTTCGAGATAAAATTCATG 3' |
| qCaHsp27.6 | 5' AATGAAACTGCAACAGAAGCTC 3' | 5' CATCCTCGGAGACCATTCAC 3' |
| qCaHsp28.4 | 5' CAAGAAACCAGCGGAAGAG 3' | 5' TGTGGCTTGTCAGCATTAGTC 3' |
| qCaHsp30.1 | 5' CGGGGATTCTTGGTACTGG 3' | 5' TGTTTCTTGGCTCACTTGTTCT 3' |
| qCaHsp35.5 | 5' GATGGAAATGGGACTCAAAC 3' | 5' GCCAGCTCTGTCTTTTGC 3' |
